# Multiparametric imaging with heterogeneous radiofrequency fields

**DOI:** 10.1038/ncomms12445

**Published:** 2016-08-16

**Authors:** Martijn A. Cloos, Florian Knoll, Tiejun Zhao, Kai T. Block, Mary Bruno, Graham C. Wiggins, Daniel K. Sodickson

**Affiliations:** 1 Bernard and Irene Schwartz Center for Biomedical Imaging, Center for Advanced Imaging Innovation and Research, Department of Radiology, New York University School of Medicine, 660 1st Avenue, New York, New York 10016, USA; 2 Siemens Medical Solutions USA Inc., 40 Liberty Boulevard, Malvern, Pennsylvania 19355, USA

## Abstract

Magnetic resonance imaging (MRI) has become an unrivalled medical diagnostic technique able to map tissue anatomy and physiology non-invasively. MRI measurements are meticulously engineered to control experimental conditions across the sample. However, residual radiofrequency (RF) field inhomogeneities are often unavoidable, leading to artefacts that degrade the diagnostic and scientific value of the images. Here we show that, paradoxically, these artefacts can be eliminated by deliberately interweaving freely varying heterogeneous RF fields into a magnetic resonance fingerprinting data-acquisition process. Observations made based on simulations are experimentally confirmed at 7 Tesla (T), and the clinical implications of this new paradigm are illustrated with *in vivo* measurements near an orthopaedic implant at 3T. These results show that it is possible to perform quantitative multiparametric imaging with heterogeneous RF fields, and to liberate MRI from the traditional struggle for control over the RF field uniformity.

## Abstract (web summary)

Magnetic resonance fingerprinting (MRF) requires a uniform B_1_^+^ radiofrequency field. Here the authors present plug-n-play MRF, a technique that enables multiparametric imaging with heterogeneous B_1_^+^ fields, and demonstrate its utility in the presence of RF distortion caused by a metallic orthopaedic implant.

## Graphical abstract

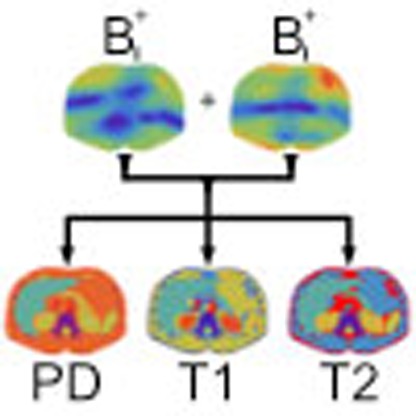

During the last four decades, magnetic resonance imaging (MRI) has evolved into a pre-eminent clinical imaging modality and invaluable research tool. Much of this development proceeded through increasingly advanced mechanisms to subdue experimental imperfections within the magnetic resonance (MR) scanner. MR systems now deploy ever-more-elaborate calibration^1^,^2^,^3^,^4^ and compensation mechanisms^5^,^6^,^7^,^8^,^9^,^10^ in pursuit of an elusive balance between image quality and scan time. Nevertheless, most clinical MR examinations have noticeable imperfections, and experienced radiologists are called upon to see through the subtle distortions produced by suboptimal conditions^11^,^12^,^13^,^14^. One notable source of imperfections is inevitable spatial variations in the radiofrequency (RF) fields used to excite and detect the MR signal.

Far from being a new problem, non-uniform RF fields have posed a challenge since the inception of MRI. Applied RF fields (known as B_1_^+^ fields) illuminate the subject and determine the ‘exposure' of the image^5^. A non-uniform exposure can result in undesirable artefacts that shade the field of view and obfuscate important details in the image^11^,^12^,^13^,^14^. Attempts to overcome these artefacts generally strive to harness B_1_^+^ to best approach a uniform exposure. However, subject-specific electrodynamic interactions between the incident RF field and patient anatomy preclude the construction of a tractable general-purpose uniform B_1_^+^ source^15^. Instead, a large body coil built into the bore of the scanner is often used to approximate an even illumination^16^,^17^. Residual imperfections are then mitigated using dedicated compensation techniques such as adiabatic pulses^5^, whose efficacy is limited by safety constraints on RF energy deposition into tissue, as well as by unwanted relaxation and magnetization transfer effects^18^. Alternatively, more complex techniques, such as parallel transmission with multiple RF sources, can be employed to tailor the net illumination to the subject^6^,^7^. However, such tailored solutions require meticulous calibration measurements^1^,^2^,^3^,^4^ and elaborate inline calculations^6^,^7^,^19^,^20^ that impede workflow and lengthen examination time^20^,^21^.

Here we eliminate the need for a uniform B_1_^+^ field, adiabatic pulses, dedicated calibration measurements and subject-specific calculations with a solution we call ‘Plug-and-Play MR fingerprinting' (PnP-MRF). Like other techniques before it, the MRF approach was originally developed and applied in the traditional context of a precisely calibrated and uniform B_1_^+^ field^22^. PnP-MRF liberates MRF from these constraints and enables accurate quantitative imaging free of RF-related artefacts. At the same time, PnP-MRF extracts the actual B_1_^+^ fields experienced by the imaged body from the measurement data. This self-calibrating nature of our method marks a clear departure from the traditional paradigm of calibration and control in MRI, and enables use of the method without fine-tuning, that is, in a ‘plug-and-play' mode, over a broad range of experimental conditions.

## Results and Discussion

### The impact of B_1_^+^ non-uniformities on MRF

MRF measurements are designed to probe the dynamics of a spin system as it is perturbed by a sequence of RF and gradient pulses. Instead of encoding individually intelligible images, MRF seeks to capture the time evolution of the net magnetization in each voxel as it evolves throughout the sequence. When the B_1_^+^ field is perfectly uniform, each of these signal evolutions, referred to as fingerprints, can be matched uniquely to one entry of a simulation-based dictionary, which identifies underlying tissue properties, including relative proton spin density (PD), longitudinal relaxation time (T1) and transverse relaxation time (T2).

**Figure 1:**
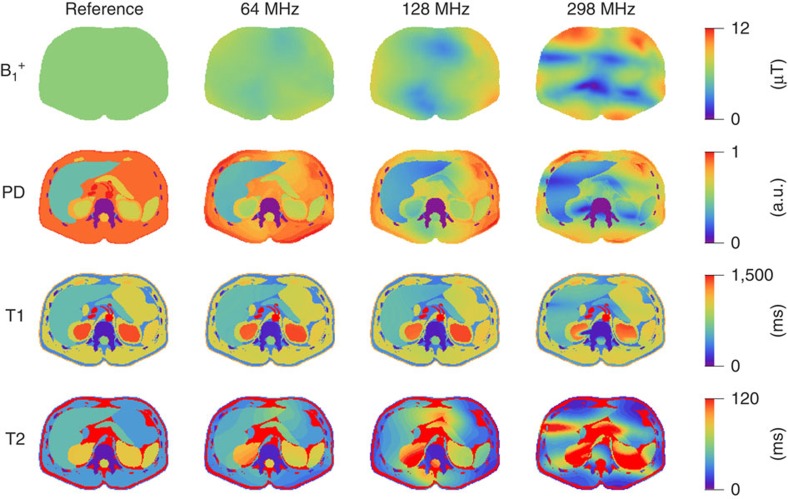
The performance of the conventional MRF framework at different field strengths. Each sub-figure shows the same axial slice through the abdomen. The top row shows simulated B_1_^+^ field distributions produced by a bore-sized circularly polarized RF body coil at three different proton Larmor frequencies associated with 1.5 T (64 MHz), 3.0 T (128 MHz) and 7.0 T (298 MHz) MR scanners. The left-most reference case assumed a (physically infeasible) fully uniform B_1_^+^ field. The CV of the | B_1_^+^| field amplitudes was 0, 6, 24 and 39%, for the reference, 64, 128 and 298 MHz cases, respectively. The next three rows show the PD, T1 and T2 maps reconstructed from a synthetic MRF measurement at each field strength, juxtaposed to the ground truth maps (left column) used as inputs to the simulation (based on assignment of representative parameter values for distinct tissue types in each segmented image). For simplicity of comparison, the T1 and T2 values were kept constant with field strength. Note the progressive deviation from ground truth with increasing frequency.

However, MR systems seldom produce a uniform B_1_^+^ field. The top row in Fig. 1 shows the simulated B_1_^+^ field distributions produced by an idealized 16-rung bore-sized transmit coil at different frequencies. Even at the lower end of the clinical spectrum (1.5 T, 64 MHz), where the RF wavelength is still larger than the axial cross-section of the average human torso, there are noticeable spatial variations in the B_1_^+^ field. Although the coefficient of variation (CV) of the |B_1_^+^| field is only 7%, this variation already biases the T2 and PD maps measured using MRF (Fig. 1, column 2).

As the frequency is increased from 64 to 128 MHz (3.0 T), the RF wavelength becomes comparable to the abdominal cross-section, which gives rise to strong B_1_^+^ depressions in the anterior and posterior areas of the body (Fig. 1, column 3). This characteristic B_1_^+^ distribution (CV=24%), often observed at 3.0 T (refs 11, 12), further degrades the PD and T2 maps to the point where some of the T2 values measured in the right kidney exceed the scale set by the maximum values included in the ground truth simulation.

At 298 MHz (7.0 T), the RF wavelength is reduced to ∼15 cm, which causes the aforementioned B_1_^+^ depressions to extend into so-called B_1_^+^ voids (CV=39%), that is, areas that do not experience any RF excitation (Fig. 1, column 4). In the absence of any appreciable B_1_^+^, even adiabatic pulses, such as the one at the start of the conventional MRF sequence^22^, fail to yield reliable inversions (Supplementary Fig. 1). As a result, in addition to strong distortions in the T2 and PD maps, the reconstructed T1 map now also shows strong artefacts (Fig. 1, column 4).

### Multiparametric imaging with heterogeneous RF fields

**Figure 2:**
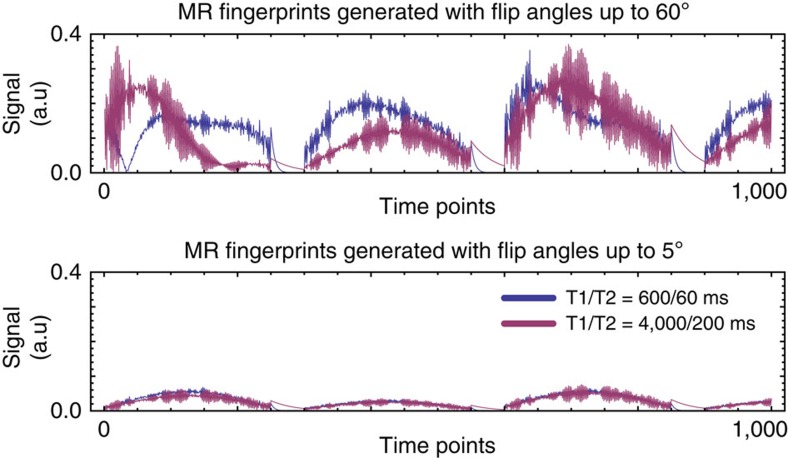
MR fingerprints produced with different _1_^+^ amplitudes. The top graph shows the signal evolution (MR fingerprint) of two different tissues. Tissue one (shown in blue) has a T1 of 600 ms and a T2 of 60 ms. Tissue two (shown in purple) has a T1 of 4,000 ms and a T2 of 200 ms. When the train of RF pulses induces relatively large flip angles (up to 60 degrees), two distinct fingerprints are obtained. The bottom graph shows again the signal evolution of the same two tissues when the train of RF pulses is constrained to the small-tip-angle domain (up to 5 degrees), similar to what may happen in areas with low B_1_^+^. In this situation, the two fingerprints lose their unique features and become signal-starved, which makes it nearly impossible to tell them apart.

While it has been shown that a dedicated^23^ or integrated^24^ B_1_^+^ calibration can be used to correct mild B_1_^+^ distortions in MRF measurements, it is impossible to extract a viable signal from areas devoid of RF field. MR fingerprints originating from such regions are signal-starved and governed by small-tip-angle excitations, which limits the signal encoding to the linear domain of the Bloch equations^25^. As a result, the MR fingerprints belonging to different tissues become indistinguishable (Fig. 2).

**Figure 3:**
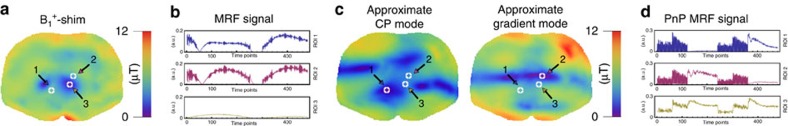
_1_^+^ field distributions at 7 T and corresponding MR fingerprints. The same three regions of interest are marked in each B_1_^+^ map. Each region of interest falls in a distinct B_1_^+^ void in one of the maps. (**a**) The B_1_^+^ distribution obtained in the abdomen after RF shimming with 16 azimuthally distributed dipoles (CV=23%). (**b**) The first 480 time points of the MR fingerprint measured in the three different regions of interest resulting from excitation with the RF-shimmed B_1_^+^ distribution. The fingerprint in the region of the B_1_^+^ void (3) is signal-starved and notably less distinctive than the other two fingerprints (obtained from regions 1 and 2). (**c**) The B_1_^+^ distribution of the approximate CP mode obtained by aligning the MR signal phases in the centre of the abdomen and the approximate gradient mode derived by incrementing the RF phase according to the azimuthal angle of the coil element (CV=41% and 38%, respectively). (**d**) The first 480 time points of the PnP-MR fingerprint measured in the three different regions of interest resulting from interleaved excitations using the two coil modes. Since the B_1_^+^ nulls for the two modes do not overlap, all regions experience substantial excitation at regular intervals during the PnP-MRF sequence, and all three fingerprints retain high signal and distinctiveness.

**Figure 4:**
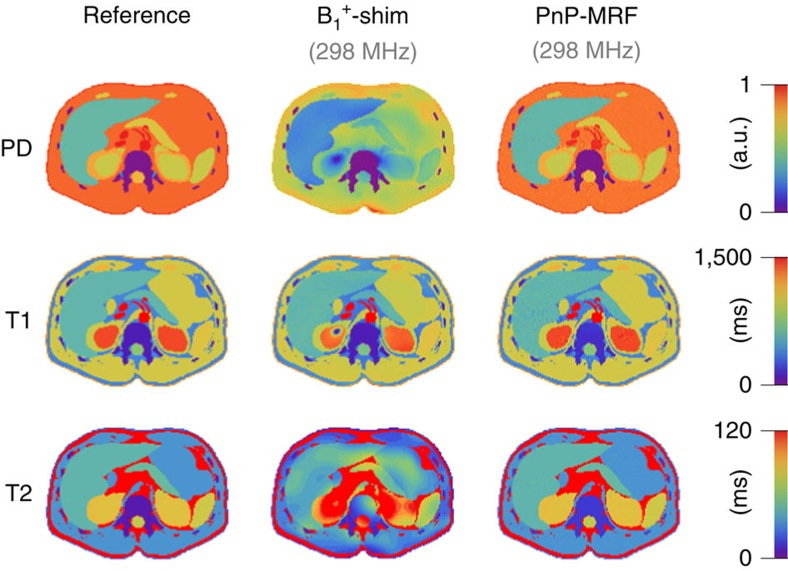
_1_^+^-shimmed MRF versus PnP-MRF. The left-hand column shows the ground truth abdominal PD, T1 and T2 maps. The middle column shows tissue property maps reconstructed from a synthetic 16-channel B_1_^+^-shimmed conventional MRF experiment at 7 T. The right-hand column shows tissue property maps reconstructed from a synthetic PnP-MRF experiment interweaving the approximate CP and gradient mode at 7 T. Each row of figures is plotted with the same scale. Whereas residual B_1_^+^ variation in the shimmed case results in substantial artefacts (particularly in the T2 and PD maps, but also in selected regions of the T1 map—for example, in the right kidney), the PnP-MRF case shows no systematic deviation from ground truth.

One strategy to mitigate such signal voids is B_1_^+^ shimming, which strives to create a desirable B_1_^+^ distribution in the subject by driving multiple independent RF sources in parallel. The above-mentioned body coil, for example, can be broken up into 16 azimuthally distributed dipole elements, which allows the field non-uniformity to be reduced from a CV of 39% to a CV of 23% at 298 MHz (Fig. 3a). Although this is a substantial improvement, it is simply impossible to create a truly uniform B_1_^+^ field with a finite number of RF sources^15^. Owing to the short RF wavelength in human tissue, an interference pattern forms, which often leads to residual B_1_^+^ voids in the abdomen (Fig. 3b) and artefacts appear in the derived multiparametric maps (Fig. 4, centre column).

PnP-MRF circumvents these problems by co-encoding the B_1_^+^ distribution into the MR fingerprints. This way, the effect of spatial variations in the B_1_^+^ fields can be separated out and quantified alongside tissue parameters of interest, in one comprehensive image reconstruction process. Moreover, PnP-MRF enables multiple complementary RF configurations to be interwoven into the sequence, which circumvents the usual pitched battle against B_1_^+^ voids.

Instead of trying to calibrate out all B_1_^+^ voids, a ‘plug-and-play' solution can be constructed by interweaving two or more distinct coil modes into the MRF framework. Even though the exact B_1_^+^ distributions are not known *a priori*, different coil configurations tend to have distinct features. The CP mode (a bright centre surrounded by a halo of B_1_^+^ voids) and the Gradient mode (which contains a RF void in the centre surrounded by areas with relatively high B_1_^+^) of a coil array (Fig. 3c), for example, have been noted to be largely complementary^26^. Experimentally, approximations of these modes can be identified in less than 10 s by first aligning the MR signal phases in a central region of the subject to obtain the approximate CP mode from which the approximate gradient mode can be derived based on the azimuthal angle of the coil elements. Moreover, once the system has been characterized, these coil modes can be hardcoded into the sequence for future use.

Although each of these approximate coil modes produces a heterogeneous B_1_^+^ field (CV 41% and 38%, respectively), a viable MR fingerprint is obtained from all regions in the field of view because the B_1_^+^ voids do not overlap (Fig. 3d). This makes it possible to reconstruct artefact-free multiparametric maps throughout the abdomen at 7.0 T (Fig. 4, right column).

This new paradigm of interweaving multiple heterogeneous RF fields has the potential to overturn the fundamental design considerations governing transmit coils in MRI. Instead of striving to realize coil configurations that minimize RF field imperfections, the focus can be shifted towards designs that provide two or more complementary illuminations. This is an inherently easier problem to tackle because, in our approach, RF pulses are played out in an interleaved manner and the illuminations do not directly interact with one another. Thus, in contrast to tailored field configurations in traditional MRI techniques (B_1_^+^ shimming^15^ or parallel RF transmission^6^,^7^), here the destructive interference between RF sources is completely avoided. Importantly, such an approach also avoids the constructive interference of electric fields that could cause local heating of conductive tissues^27^,^28^ and does not require energetic refocusing or adiabatic pulses. In other words, our approach addresses the two greatest hurdles for widespread adoption of multicoil transmission: safety and workflow.

### Experimental comparison at 7 T

**Figure 5:**
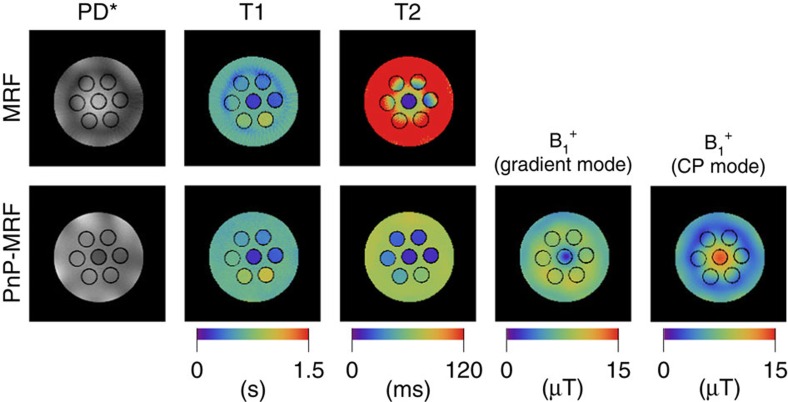
Experimental results obtained at 7 T. The top row shows the multiparametric maps (PD, T1 and T2) obtained with the conventional MRF sequence. The bottom row shows the multiparamatric maps (PD, T1, T2 and B_1_^+^) obtained with our PnP-MRF approach. The asterisk indicates that no receive sensitivity correction was applied. All subfigures show the same axial slice through a 16 cm diameter cylindrical phantom.

The same wavelength effects that complicate abdominal imaging can also be observed in smaller samples with a high relative permittivity. A water phantom at 7 T, for example, also gives rise to extremely heterogeneous B_1_^+^ fields. The top row in Fig. 5 shows the experimental results obtained with the MRF method in a 16.5 cm diameter phantom at 7 T (CV of the B_1_^+^ is 39%). Similar to the numerical experiments shown above (Fig. 1), the T1 map remains accurate in most places except for those areas where the B_1_^+^ is extremely low and the adiabatic condition of the preparation pulse cannot be fulfilled. The T2 map, on the other hand, is biased throughout the slice except for the central area where the B_1_^+^ amplitude best approximates the nominal flip-angle for which the sequence was designed.

The bottom row in Fig. 5 shows the multiparametric maps measured using PnP-MRF. Analogous to the PnP-MRF simulations shown in Fig. 4, we first obtained the approximate CP mode by aligning the phases in the centre of the phantom and derived the approximate gradient mode based on the azimuthal angle of the coil elements. This pre-scan adjustment can be performed in 7 s with little or no impact on the workflow. Moreover, this calibration only needs to be performed once for each coil and can then be hardcoded into the PnP sequence.

Even though the B_1_^+^ distributions produced by these same coil modes look fundamentally different from those in the abdomen (Fig. 4c versus Fig. 5), the fields produced by these two coil configurations remain complementary. Interwoven into the PnP-MRF sequence these two coil modes circumvent persistent RF voids, which enables the accurate quantification of PD, T1, T2 and B_1_^+^ throughout the slice.

Currently, B_1_^+^ maps are not used in routine clinical imaging. Nevertheless, these maps capture the complex interactions between the subject anatomy and the incident RF field. The opportunity to rapidly quantify the RF field could be an asset for electrical property tomography, an MR technique that aims to recover dielectric tissue property distributions based on the local curvature of B_1_^+^ (refs 29, 30). In turn, these dielectric maps could provide a new set of complementary parameters (tissue conductivity and permittivity) to aid in the diagnostic process^31^,^32^.

Instead of the simultaneous estimation of B_1_^+^ fields and tissue properties shown here, one could envision the use of dedicated traditional B_1_^+^calibration scans as prior information for PnP-MRF. Although prior knowledge of the B_1_^+^ field distribution would help to constrain the cross-section of the dictionary that needs to be considered during the reconstruction process, any inaccuracies in those external B_1_^+^ maps will propagate into the T1 and T2 maps. Considering that most traditional B_1_^+^ mapping techniques are only accurate within a relatively limited range of B_1_^+^ values, residual artefacts may be expected when imaging with highly heterogeneous RF fields (Supplementary Note 1 and Supplementary Fig. 2). Moreover, external B_1_^+^ calibration scans are prone to misregistration artefacts because of subject motion, and they require additional scan time that increases the duration of the comprehensive examination.

### Imaging near orthopaedic implants

**Figure 6:**
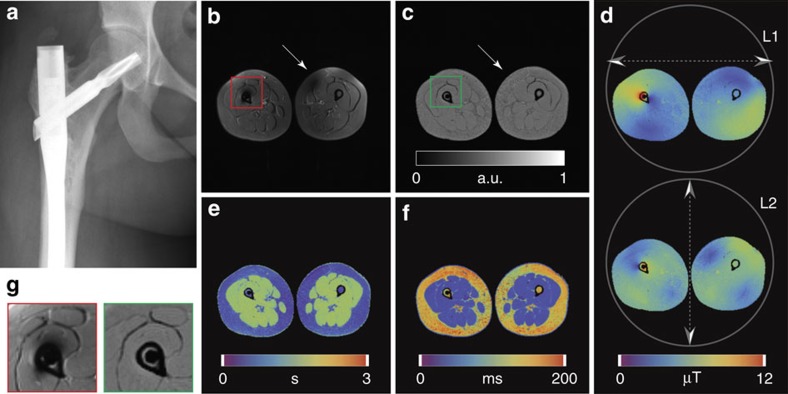
Conventional MRI versus PnP-MRF in the presence of an orthopaedic implant. (**a**) X-ray image showing the orthopaedic implant. (**b**) Contrast-weighted axial image through the legs obtained using a conventional inversion recovery (IR) TSE sequence. Signal voids appear both in the vicinity of the implant and in the contralateral leg. (**c**–**f**) Quantitative maps obtained using PnP-MRF, including PD, B_1_^+^, T1 and T2, respectively. Units are arbitrary for PD (**c**), micro Tesla for B_1_^+^ (**d**), seconds for T1 (**e**) and milliseconds for T2 (**f**). (**g**) Enlargements, extracted from **b** (red frame) and **c** (green frame), comparing the region surrounding the implant with conventional (left) and PnP-MRF (right) approaches. (PnP-MRF scan time: ∼28 s per slice.) Note the absence of B_1_^+^-related signal voids in any of the PnP-MRF parameter maps.

The previous experiments focused on the feasibility of quantitative imaging at ultrahigh field strength (7 T, 289 MHz)—an area that remains highly challenging despite many years of research and optimization using traditional paradigms. This, however, is just one example out of a large body of documented cases describing subject-specific RF field interactions that lead to severe artefacts in MR^11^,^12^,^13^,^14^,^15^,^20^,^21^,^33^. Orthopaedic implants, such as the titanium rod depicted in Fig. 6a, for instance, interact strongly with the incident RF field, leading to significant B_1_^+^artefacts at clinical field strengths^14^.

To demonstrate the feasibility of our PnP-MRF approach in a clinically relevant setting, we implemented it on a whole-body 3 T (128 MHz) MRI system (Skyra timTX, Siemens, Erlangen, Germany), in which the built-in transmit coil can be decomposed into two linear components (L1 and L2). We used this setup either (a) to drive both linear components simultaneously with a fixed amplitude and phase relationship to produce an elliptically polarized (EP)-mode (a two channel B_1_^+^ shim) optimized for the anatomy under investigation^17^ or (b) to interleave the two linear components one after the other into the PnP-MRF sequence.

The appearance of B_1_^+^ artefacts depends on the level of B_1_^+^ non-uniformity as well as on the imaging sequence selected. Clinical MR examinations primarily use turbo spin echo (TSE)-based sequences especially near implants because of their high signal-to-noise ratio, their flexible contrast weighting and their inherent robustness against variations in the main magnetic field (*B*_0_). Here we compare PnP-MRF to an inversion recovery (IR) TSE sequence, which provides one additional parameter, the inversion time, to adjust the contrast weighting. We used this flexibility to create an intuitive visual comparison by adjusting the inversion time to create a contrast weighting which resembles the PD map obtained with PnP-MRF.

In this case, the B_1_^+^ heterogeneities obscure tissues surrounding the implant and shade part of the opposing leg in the IR-TSE image (Fig. 6b). The PD map obtained with our PnP-MRF framework, on the other hand, clearly depicts all tissues throughout the field of view (Fig. 6c). Although both modes of the coil (L1 and L2) interact with the rod (CV=36 and 22% for the first and second transmit channel, respectively), their B_1_^+^ profiles remain complementary (Fig. 6d). Consequently, each location is adequately illuminated by at least one of the modes, which allows accurate artefact-free quantitative maps to be reconstructed in situations where traditional contrast-weighted MR techniques struggle (Fig. 6e,f). Shading due to RF variations no longer overshadow the bone marrow and muscle tissues adjacent to the implant (Fig. 6g) and RF-related inhomogeneities affecting the opposing leg are also removed (Fig. 6b vs c). The IR-TSE parameters used here produce a relatively bland contrast, which helps to visualize the B_1_^+^ artefacts. Nevertheless, the same artefacts can also be observed in a more standard PD-weighted TSE image (Supplementary Fig. 3).

In some less-challenging cases, a single inhomogeneous RF field may suffice to perform a PnP-MRF measurement. For example, in the absence of an orthopaedic implant, the exposure produced in the legs by the CP or EP mode, although heterogeneous, is often free of B_1_^+^ voids (Supplementary Fig. 4). In such cases, either of these modes may serve as a single heterogeneous field source, since interleaved illumination by L1 and L2 is no longer required to ensure adequate exposure in all regions of interest (ROI). As in the multi-illumination implementation described above, the non-uniform B_1_^+^ distribution produced by such a source is distilled from the measurement data, leaving a complete set of unbiased multiparametric maps without need for additional calibration.

Besides heterogeneous B_1_^+^ fields, main magnetic field variations are another long-standing problem in MR. It has already been shown that MRF is relatively robust against such field variations^22^,^34^. Nonetheless, in extreme cases, e.g., near stainless steel implants as opposed to the titanium implant shown here, additional work may be necessary to avoid residual artefacts. In particular, deformation of the excitation slice profile may become a problem, which could be addressed by switching to a three-dimensional (3D) image-encoding strategy.

One other noteworthy observation is the lack of contrast in the PD map obtained with our PnP-MRF method (Fig. 6c). Contrary to many clinical PD-weighted imaging protocols (see, for example, Supplementary Fig. 3), our PD map suggests that the total number of protons per unit of volume is similar in adipose and muscle tissues (Supplementary Note 2 and Supplementary Fig. 5). Specialized MR techniques such as (refs 35, 36) and mass spectrometry data indeed support the notion that adipose and muscle tissues have a comparable proton density (see Supplementary Note 3). However, hidden beneath this bland PD map lies another contrast-rich landscape that can be accessed by incorporating the Dixon fast-water separation method^35^ into the PnP-MRF framework (Supplementary Note 4).

### Validation

**Figure 7:**
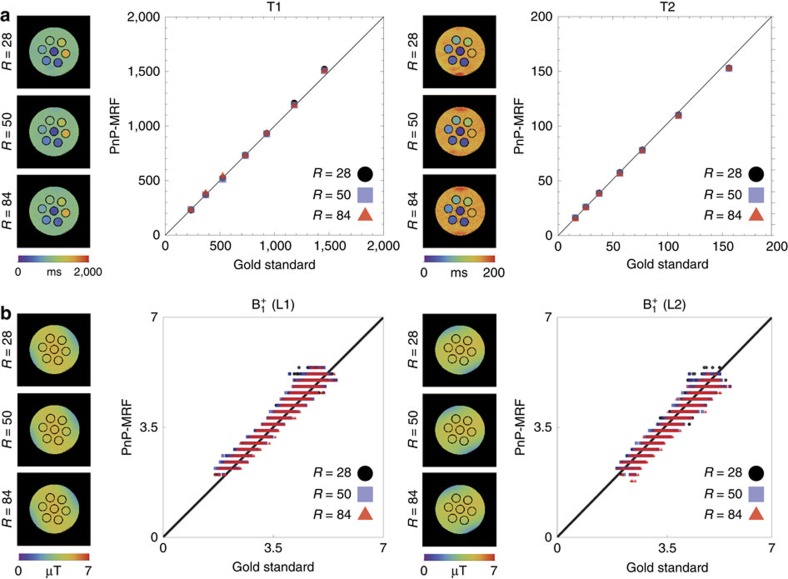
Phantom validation of PnP-MRF. (**a**) The mean T1 (left) and T2 (right) value (in milliseconds) across each of the seven sample tubes (cross-section images shown to the left of each plot) comparing PnP-MRF at three different acceleration factors (*R*) with values from a separate 5.5-h gold-standard measurement (maps not shown). (**b**) Scatter plots of B_1_^+^ field amplitudes (in μT) across the phantom measured using PnP-MRF at different acceleration factors (B_1_^+^ maps shown to the left of each plot) as compared with values from a separate 3 min 50 s gold-standard measurement (maps not shown). In all cases, a high degree of correspondence may be noted between PnP-MRF measurements and time-consuming dedicated gold-standard measurements.

Quantitative results from *in vivo* measurements in relation to literature values are summarized in Supplementary Table 1. To further validate PnP-MRF, phantom measurements were compared against a clinically prohibitive 5.5-h gold-standard measurement. Full sampling each time point in the MR fingerprint would require a long time. Instead, the acquisition is accelerated by undersampling each exposure. As described in ref. 22, we also rely on the dictionary-matching process to filter out the resulting spatial aliasing. Three different acceleration factors *R*={28, 50, 84} were evaluated, corresponding to a total scan time of 21, 12 and 7 s per slice, respectively. As shown in Fig. 7a, excellent correspondence was maintained over a broad range of physiological values (T1<2 s and T2<150 ms), with little or no change between *R*=28 and *R*=50 (Supplementary Table 2). Although there is some residual variation (s.d. ≈5% or less) within each ROI (Supplementary Table 2), precision estimates, derived from 50 repeated measurements, show that the average over a small (100 voxel) ROI is highly reproducible leading to less than 2% variation across all measurements. (Supplementary Fig. 6).

However, with current proof-of-principle implementation, it still remains difficult to measure extremely long T2 values in areas with a low signal-to-noise ratio. This can be understood by looking at the sequence design (Supplementary Fig. 7). Currently, the RF pulse train is segmented into groups of 120 excitations (cumulative duration ≈600 ms), which limits the ability to differentiate fingerprints with a very long T2. The adipose tissue in Fig. 6f, for example, shows some T2 variability that may be related to the multiexponential relaxation of the fat in combination with variations in the signal-to-noise ratio. Longer RF train segments could help make fingerprints with a long T2 more distinct, thereby reducing the sensitivity to noise.

In addition to the tissue-dependent parameters T1 and T2, we also validated the B_1_^+^ distributions (CV=15 and 16% for the first and second transmit channels, respectively). Results of a dedicated saturation-based RF field mapping calibration measurement (total scan time 3 min 50 s per slice)^3^ were compared with the B_1_^+^ maps recovered during the PnP-MRF reconstruction process (Fig. 7b). This test confirmed that our approach indeed extracts the correct B_1_^+^ contributions from the measurement (correlation factors exceed 0.96 at all acceleration factors). In other words, PnP-MRF exhibits a self-calibrating property that allows quantitative MR imaging with highly non-uniform RF fields.

One important asset of MRF is its efficiency, that is, the ability to rapidly quantify multiple tissue properties at once. Table 1 lists the scan time per slice from two prominent publications on MRF, juxtaposed to our PnP-MRF experiments. Although PnP-MRF simultaneously quantifies B_1_^+^ in addition to the tissue properties, the relative efficiency remains comparable, especially considering that the times listed for conventional MRF do not include the time that might be required for a separate B_1_^+^ calibration measurement (at least in the cases of mild heterogeneity for which B_1_^+^ correction is possible in the first place). Moreover, the joint encoding of tissue properties and B_1_^+^ information in PnP-MRF avoids potential problems related to misregistration between separate scans, and provides a natural mechanism to circumvent B_1_^+^ voids.

In a sense, although it departs from long-standing conventions of MR image acquisition, the PnP-MRF approach to embracing rather than controlling inhomogeneities represents a natural extension of the fundamental principles upon which MRI was first founded. In their seminal 1973 articles, Lauterbur^37^ and Mansfield and Grannel^38^ showed that high-resolution images could be created using induced local interactions with heterogeneous magnetic fields—fields that might otherwise have been viewed as imperfections in traditional MR experiments. Their disruptive invention, however, still required that the image-encoding fields be known in advance. In the current work, we have demonstrated a means of self-discovering, from a single suitably encoded data stream, diverse and accurate information not only about the imaged body but also about the conditions under which images have been generated. Although the method described here is specific to MRI, it is based on a broad underlying fundamental concept of treating heterogeneities in the experimental conditions as a source of information rather than as a generator of artefacts, and leveraging complementary heterogeneities for robust encoding of information.

## Methods

### Sequence design

The design space for MRF sequences is quite broad, given the large number and variety of excitations and delays involved^22^. In the absence of an analytic sequence optimization process, we designed a proof-of-principle sequence.

The PnP-MRF sequence consists of four segments, each containing 120 excitations separated from one another by a 4.8–8 ms interval (Supplementary Fig. 7). The first and third segments contain spoiled gradient echoes, which alternate, from one echo to the next, between two orthogonal coil configurations. (In general, an arbitrary number of complementary RF coils or coil configurations may be used.) These RF-spoiled excitations combine the cumulative effects of both RF coil configurations into a complex spin evolution and predominantly serve to encode B_1_^+^ and T1. The other segments, with 0/180 RF phase cycling and spoiling gradients, allow stimulated echoes to form, adding a T2 relaxation component into the mix. Collectively, these 480 excitations (4 segments × 120 per segment) capture a distinct signal evolution. This signal evolution acts like a ‘fingerprint', which is used to identify the underlying tissue properties^22^.

In addition to a spatially varying B_1_^+^ amplitude, each excitation also has its own spatially varying phase distribution (defined as the argument of the complex B_1_^+^ field). Because the relative phase between excitations contains no clinical information, we designed the sequence such that the relative B_1_^+^ phase distributions are not entangled with the spin dynamics. In the first and third segments, the phase distribution produced by the preceding excitation is removed by de-phasing the transverse magnetization with a combination of gradient and RF spoiling. The other segments, however, need to partially refocus the transverse magnetization induced by preceding excitations in order to encode T2. Although it is still possible to interweave multiple RF coil configurations, this would entangle the relative transmit phase between excitations into the fingerprint, which would expand the size of the database required for fingerprint matching. Instead, we simply dedicate a single RF coil configuration to each of these two segments (Supplementary Fig. 7). This way, even if an RF void exists in one of the illuminations, the fingerprints remain viable, provided that the other illumination does not have an RF void in precisely the same location.

To quantify T1 and T2 accurately, the fingerprint must sample the spin dynamics over a time interval comparable to (or exceeding) the longest expected relaxation time. Instead of acquiring one long continuous fingerprint^22^, a delay time (Δ*t*) is inserted between the segments during which the magnetization is allowed to partially recover (Supplementary Fig. 7). This has three advantages. First, it strongly encodes T1 without depositing additional RF energy into the sample. Second, it allows the longitudinal magnetization to regrow, which translates to larger signal amplitudes in later sections of the fingerprint. Third, this time can be used to measure additional segments corresponding to two more slices. At 3 T, most human tissues cannot relax completely during this delay. Consequently, the spin evolution during each segment is dependent on the previous segment, that is, all four segments collectively form one MR fingerprint that simultaneously identifies the underlying B_1_^+^ distributions and tissue properties (PD, T1 and T2).

The second and fourth segments contain larger driving voltages to emphasize the refocusing component. The exact relative driving voltages and phase increments can be found in the Supplementary Data 1. Before each examination, the relative drive voltages are scaled by the inline RF transmit adjustment available on all MR systems. Owing to the relatively large dynamic range of driving voltages in the sequence, this need not be precise. When using a 2 ms sinc pulse with a time-bandwidth product of 3, a peak B_1_^+^ anywhere between 3 and 12 μT in either illumination is sufficient to produce accurate multiparametric maps (Fig. 6c–f).

### Spatial encoding and intermediate image reconstruction

Fully sampling all 480 time points with a clinically acceptable matrix size *M* × *M* would result in an impractically long scan time. Instead, we only acquire a small number (*N*) of radial samples (spokes). Traditionally, the use of such an extreme acceleration factor (*R*), as defined in equation 1, would result in insurmountable streak-like aliasing artefacts. In the MRF framework, however, these incoherent artefacts add a noise-like modulation to the fingerprint, which has a relatively benign effect on the reconstruction process^22^.


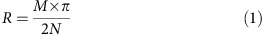


Each time point in the fingerprint is sampled with *N* evenly distributed radial spokes (Supplementary Fig. 8a). To emphasize the incoherence between aliasing artefacts, the readout is rotated by 14 × 6/*N* degrees between excitations. This ensures that subsequent time points cover complementary regions of k-space (Supplementary Fig. 8b). In turn, this enforces a different distribution of streak-like artefacts between exposures in the image domain (Supplementary Fig. 8c). The images from which the fingerprints are extracted are reconstructed using a non-uniform fast Fourier transform^39^ augmented with parallel imaging^40^,^41^,^42^,^43^. The incorporation of a parallel imaging strategy is optional, since the matching process itself can filter out most aliasing (see also Supplementary Note 5 and Supplementary Table 3). The image reconstruction process was implemented in MatLab (The MathWorks Inc., Natick, MA, USA).

### Dictionary construction

The database with simulated MR fingerprints, hereafter referred to as the dictionary, was computed based on the extended-phase graph formalism^44^. For the *in vivo* and *in vitro* experiments (as opposed to the *in silico* experiments), the slice profile was incorporated into the simulation based on the Fourier transform of the RF waveform. The final fingerprint is the sum of the different contributions under the slice profile after each excitation. The extended-phase graph simulation software was developed in-house and was written in C++.

The final dictionary has four dimensions: T1, T2 and B_1_^+^ amplitude for each of the two transmit channels. (The PD is derived from the ratio between the measured and simulated fingerprints.) Because of the high dimensionality of this space, the number of values along each axis needed to be constrained. To maintain constant relative accuracy, we incremented the T1 and T2 values in steps of 5%. T1 values ranged from 150 to 4,564 ms, and T2 values ranged from 15 to 456 ms. B_1_^+^ values were evenly distributed between 0 and 15 μT in increments of 0.2 μT. The database was compressed, as described in the next section, and was permanently stored. In total, the dictionary contained over 10^7^ entries, and was ≈3 GB in size (after compression). Using a four-core laptop computer running at 2.4 GHz, the approximate calculation time for the dictionary was ≈2 h without the slice profile, and ≈10 h when the slice profile was included. Note that this pre-computation step is performed only once. It has no impact on the reconstruction time or scan duration, and it can be used to reconstruct all future measurements.

### Fingerprint compression

Both simulated and measured fingerprints were compressed before dictionary matching. This compression step serves two purposes. First, it accelerates the matching process by reducing the number of data points in each fingerprint. Second, it alleviates computer memory constraints by reducing the size of the dictionary. Both of these two properties are particularly useful in the context of PnP-MRF, where each additional independent RF source adds one more dimension to the dictionary.

McGivney *et al.*^45^ demonstrated a mathematically rigorous MR fingerprint compression framework using the singular value decomposition (SVD). However, unlike conventional MR fingerprints, a PnP-MR fingerprint contains signals generated with different transmit channels. The SVD compression can mix these signals, which would entangle the relative transmit phases into the compressed fingerprint. Instead, we implemented a heuristic fingerprint compression method based on the concept of k-space view-sharing^5^,^46^. The procedure simply integrates sets of 15 consecutive complex sample points obtained with the same RF coil configuration. After compression, each fingerprint is reduced from 480 to 32 time points (Supplementary Fig. 9). The number of projections and integration intervals were chosen based on prior experimentation, and should not be viewed as the result of a mathematically rigorous optimization process. Future work could improve on this by extending the SVD-based compression to the PnP-MRF framework.

This view-sharing-based compression can further reduce the size of the dictionary by projecting the matching processes from a complex space (phase and amplitude) on a real space. After compression, the incoherent contributions due to aliasing artefacts interfere (Supplementary Fig. 8c vs d). Under ideal conditions, and using a matched-filter coil combination to remove the relative transmit and receive phase contributions^47^, this results in a real-valued compressed fingerprint. In practice, some phase variations and residual aliasing artefacts remain (Supplementary Fig. 8d). Empirically we found that remaining phase variations can safely be disregarded by taking the absolute value.

After compression, the size of the database is reduced by a factor of 30 (the projection from complex to real values reduces the dictionary size by a factor of 2, and bundling 15 time points together reduces the dictionary size by another factor of 15). Moreover, the same compression prospectively applied to the dictionary is also applied to measured fingerprints. As noted in ref. 45, the compression can be performed in k-space directly, thus reducing the number of non-uniform Fourier transforms needed to reconstruct the final maps.

### Matching algorithm

The multiparametric maps (T1, T2 and B_1_^+^) were extracted by identifying the (compressed) dictionary element that best correlates with the (compressed) measured fingerprint. Once the best matches are found, the PD map is retrieved by calculating the ratio between the measured (un-normalized) and simulated (un-normalized) fingerprints. The system-dependent variations due to the spatial distribution of receive sensitivity profile were removed using coil sensitivity estimates derived from the central k-space portion in the first compressed time point in the fingerprint^48^.

The matching algorithm, a simple exhaustive search for the highest correlation, was implemented in C++. Using a 4-core laptop computer running at 2.4 GHz, a reconstruction time of ∼5 min per slice was obtained for a 160 × 160 voxel matrix. This optimized C++ code is freely available (including a standalone example data set) at https://bitbucket.org/macloos/pnp-mrf/wiki/Home/. If necessary, it may be possible to accelerate the matching process by re-structuring the dictionary^49^.

### Synthetic MRF and PnP-MRF experiments

Full-wave electrodynamic simulations were performed in CST Microwave Studio (Darmstadt, Germany) to obtain the B_1_^+^ distributions of a circularly polarized (CP) bore-sized transmit coil in the abdomen (Supplementary Fig. 10). The coil consisted of 16 rungs, azimuthally distributed on a 60 cm diameter cylinder ∼2 cm from the gradient shield, loaded with the Duke human body model (2 × 2 × 2 mm^3^; ref. 50). Simulations were performed at resonant frequencies of 64, 128 and 298 MHz, which correspond to the proton Larmor frequency in a 1.5, 3.0 and 7.0 T MR system, respectively.

At 298 MHz, we also extracted the B_1_^+^ profiles from each of the individual rungs in the coil. These fields were used to create a B_1_^+^ shim that strives to minimize B_1_^+^ variation throughout the slice. This was achieved by solving the following magnitude least squares optimization problem:


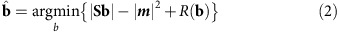


where ***S*** contains the complex B_1_^+^ distributions form each rung, ***m*** is the uniform target distribution, *R*(***b***) is an optional regularization function not used here and 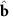 is a vector containing the optimal driving currents^51^. In this model, the arms are inside the coil and in the field of view. However, the arms are usually not of interest during an abdominal examination and were excluded from the optimization procedure and field uniformity calculations.

Instead of feeding each of the 16 rungs individual, the coil can also be driven in 16 orthogonal modes^26^. These modes (*M*_*n*_) correspond to phase combinations that are multiple of the azimuthal angle (*α*) between the coils (equation 3). In this formalism, the first mode (*M*_1_) is equal to the CP mode, which is orthogonal to the gradient mode (*M*_2_).


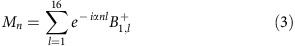


The simulated B_1_^+^ profiles were used to perform synthetic MR experiments in an axial slice through the abdomen based on the extended-phase graph formalism^44^. Each of the tissues in the body model was assigned literature T1, T2 and PD values corresponding to 1.5 T (refs 52, 53). Although T1 and T2 values are field strength-dependent, we kept the values constant to better visualize the impact of B_1_^+^ field heterogeneities on the reconstructed quantitative maps. The MRF sequence design and reconstruction were implemented as described by Ma *et al.*^22^, and the PnP-MRF sequence design and reconstruction was performed as described above using the approximate CP and gradient modes of the coil. All the codes necessary to synthesize and reconstruct these data sets are freely available at https://bitbucket.org/macloos/pnp-mrf/wiki/Home/.

### *In vivo* experiments

*In vivo* experiments were performed with a whole-body 3 T MRI system (Skyra timTX, Siemens). The built-in birdcage body coil with two drive points that correspond to the two linear modes (L1 and L2) was used for excitation. In the traditional MR framework, these are combined with a fixed phase and amplitude relation to form either the CP mode^16^ or an anatomy-optimized EP mode^17^. In our PnP-MRF framework, the two linear modes are independently interwoven into the sequence to expose the sample to complementary B_1_^+^ fields (no coil-mode calibration needed).

Axial bilateral lower-extremity images were acquired in a volunteer (25, F) with a 3 T-approved orthopaedic implant: a titanium intramedullary nail (Smith & Nephew Inc., Memphis, TN, USA) in her right femur^54^. A standard 18-channel body receive array (Siemens) was used for signal reception. The parameters for our sequence were as follows: 18 slices, TR/TE=4.8/2.3 ms, radiofrequency (RF) time-bandwidth ratio 3, RF pulse duration 2 ms, 336 × 336 matrix, 1.4 × 1.4 mm^2^ in-plane resolution, 5 mm slice and acceleration factor *R*≈44 (12 spokes per illumination, ±28 s per slice, for a total scan time of 8 min 18 s). For comparison, the product TSE sequence was used to acquire a series of different contrast-weighted images using the system default anatomy-optimized EP mode of the body coil.

To obtain a contrast weighting, which resembles the PD map obtained with PnP-MRF, we used an inversion recovery TSE with the following parameters: 18 slices, turbo factor 8, repetition time (RT)/echo time (TE)/inversion time (TI)=2,400/8.4/150 ms, 384 × 288 matrix, 1.25 × 1.25 mm^2^ in-plane resolution, 5 mm slice and total scan time ±2 min. The slices were positioned to coincide with the slices in the PnP-MRF data set.

In addition, we also obtained a PD-weighted TSE image from a separate scan session with exactly the same set-up. Sequence parameters were as follows: turbo factor 8, TR/TE=4,000/8.4 ms, 320 × 320 matrix, 1.5 × 1.5 mm^2^ in-plane resolution and 5 mm slice.

During all the experiments, the online-specific absorption rate estimation remained well below 50% of the (normal) limit. The study was approved by our institutional review board, and written informed consent was obtained before each examination.

### Phantom experiments at 3 T

Phantom experiments were performed using the same MR system described above, paired with a 20-channel head receive coil. All measurements were performed in a single session, after the phantom had acclimatized to the ambient temperature. The phantom contained seven test tubes (2.5 cm diameter), each filled with distilled water doped with different concentrations of manganese (II) chloride tetrahydrate (Cl_2_Mn 4H_2_0, Sigma-Aldrich, St Louis, MO, USA). The basin holding the samples, a 16.5 × 20.0 cm cylindrical container, was filled with a mixture of distilled water, Gadolinium (Magnevist, Bayer Healthcare, Germany) and manganese (II) chloride tetrahydrate to create a distinct background. Sequence parameters for our PnP-MRF sequence were as follows: TR/TE=4.8/2.3 ms, 18 slices, a 160 × 160 matrix, 1.5 × 1.5 mm^2^ in-plane resolution and a 5.0 mm slice thickness. The measurement was repeated three times: once with an acceleration factor of 28 (nine spokes per illumination, ±21 s per slice), once with an acceleration factor of 50 (five spokes per illumination, ±12 s per slice) and once with an acceleration factor of 84 (three spokes per illumination, ±7 s per slice). To avoid air bubbles and partial volume effects from the porous 3D printed spacers, only the centre slice was used in the validation. Each tube was manually segmented to create seven ROIs (a ±2.5 cm diameter disk matching the cross-section of each tube, ≈160 voxels).

Overnight single-spin echo experiments were performed to obtain a gold-standard T1 map (TI={25, 50, 100, 200, 400, 800, 1,600, 3,200, 6,400} ms) and T2 map (TE={12, 24, 36, 48, 60, 72, 84, 96, 144, 192, 278, 384} ms), each with a 128 × 128 matrix size. In both cases, a TR of 7.5 s was selected to minimize saturation effects (total scan time was ≈5.5 h). The gold-standard T1 was extracted from the spin echo data set using the method proposed in ref. 55. The gold-standard T2 was found by fitting an exponential to the measured data. In both cases, Mathematica (Wolfram, Champaign, IL, USA) was used to implement the fitting routine.

A pre-saturation turbo-FLASH sequence was used to obtain a set of gold-standard B_1_^+^ maps^3^. To avoid unwanted slice profile effects, a rectangular pre-saturation pulse was used. The sequence parameters were as follows: one slice, four averages, TR=10 s, inter echo time=3 ms, 128 × 128 matrix, in-plane resolution 1.5 × 1.5 mm^2^, 5.0 mm slice thickness and total scan time ±4 min. The total cross-section of the phantom, including the outer basin, was used to create the scatter plots depicted in Fig. 5b and Supplementary Fig. 11b.

On a separate day, each of the PnP-MRF measurements was repeated 50 times (for a total of 150 measurements, cumulative scan time ≈5 h). The measurements were performed in an interleaved manner, that is, one *R*=84, one *R*=50 followed by one *R*=28 measurement before starting the next repetition. This was performed to ensure that the all measurements would have a similar bias because of environmental factors such as scanner/phantom temperature drift. Each measurement was reconstructed with and without parallel imaging. For every measurement, the T1 and T2 values in each ROI (≈100 voxels ROI) were averaged to evaluate the reproducibility/precision. In addition, the histogram of all voxels in each ROI was computed.

### Phantom experiments at 7T

A 60 cm diameter bore 7 T MRI system (Magnetom 7T, Siemens) was used to compare the performance of MRF and PnP-MRF in the presence of heterogeneous B_1_^+^ fields. An in-house developed build 8-channel dipole array head-coil was used to image the same phantom as described above. Note, however, that the T1 and T2 values are expected to be different because of the increased magnetic field strength, and significantly reduced room temperature.

The conventional MRF sequence design and reconstruction method was implemented as described in ref. 34, except for the k-space sampling pattern. To avoid artefacts due to eddy currents and *B*_0_ variations, which are more prominent at 7 T, we replaced the variable density spiral sampling of k-space with a golden angle radial sampling. Five radial spokes were collected per time frame (total of 5 × 1,000 radial samples), which results in an effective undersampling factor of 20. These five spokes were acquired sequentially, allowing the spins to relax back to equilibrium (10 s) before repeating the RF pulse train. The sequence parameters were as follows: matrix size 160 × 160, 1.5 × 1.5 mm^2^ voxel size, 5 mm-thick slice and one slice. The total scan time ≈2 min.

Approximately the same total number of spokes was used in the PnP-MRF measurement (nine spokes per time frame, for a total of 9 × 480 radial samples). The sequence parameters were as follows: TR/TE=6/2.1 ms, matrix size 160 × 160, 1.5 × 1.5 mm^2^ voxel size, 5 mm-thick slice and six slices. The total scan time was ≈3 min (30 s per slice). The approximate CP and gradient coil modes used in the PnP-MRF sequence were obtained using a quick calibration scan (total scan time 7 s). This scan consists of a basic gradient echo sequence, which automatically obtains one low-resolution image for each of the eight available transmit channels. The sequence parameters for this calibration scan were as follows: TR/TE=6.8/2.5 ms, matrix size 128 × 128, 2.3 × 2.3 mm^2^ voxel size, 20 mm-thick slice, one slice. A simple Bash script installed on the host computer of the MR system extracts the different RF phases measured in the centre of the image and calculates the offset necessary to approximate the CP mode. These values are written to a file and automatically incorporated into every subsequent PnP-MRF scan.

### Data availability

The data that support the findings of this study are available from the corresponding author upon request.

### Code availability

All data and codes necessary for sequence implementation, sequence simulation, dictionary creation and image reconstruction are freely available on Bitbucket (https://bitbucket.org/macloos/pnp-mrf/wiki/Home/).

## Additional information

**How to cite this article:** Cloos, M. A. *et al.* Multiparametric imaging with heterogeneous radiofrequency fields. *Nat. Commun.* 7:12445 doi: 10.1038/ncomms12445 (2016).

## Supplementary Material

Supplementary Information Supplementary Figures 1-11, Supplementary Tables 1-3, Supplementary Notes 1-5 and Supplementary References.

Supplementary Data 1 The relative driving voltages and phase increments used in the PnP-MRF sequence

## Figures and Tables

**Table 1:** The relative time efficiency of MRF versus PnP-MRF.

Method	Matrix size	Scan time per slice (s)
MRF (Ma *et al.*^22^)	128 × 128	12
MRF (Jiang *et al.*^34^)	256 × 256	13
PnP-MRF (*R*=28)	160 × 160	21
PnP-MRF (*R*=50)	160 × 160	12
PnP-MRF (*R*=84)	160 × 160	7

MRF, magnetic resonance fingerprinting; PnP-MRF, Plug-and-Play magnetic resonance fingerprinting.

*R* indicates the undersampling factor as defined by equation 1. Matrix size is reported in voxels and scan time is reported in units of seconds per slice.
