# Supplementary material for: Multiparametric imaging with heterogeneous radiofrequency fields
Source: Nat Commun. 2016 Aug 16;7:12445. doi: 10.1038/ncomms12445 (PMC4990694; doi:10.1038/ncomms12445)
Supplement: Supplementary Information — Supplementary Figures 1-11, Supplementary Tables 1-3, Supplementary Notes 1-5 and Supplementary References. [file ncomms12445-s1.pdf]

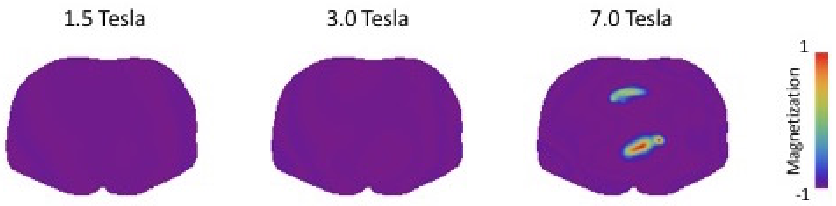

Supplementary Figure 1: Plots of the inversion fidelity produced by a high power 10ms Hyperbolic Secant adiabatic pulse using the circularly polarized birdcage coil tuned to the Larmor frequencies used at 1.5, 3.0 and 7.0 Tesla MRI. Each sub-figure shows same slice through the abdomen, plotted using the same color scale.

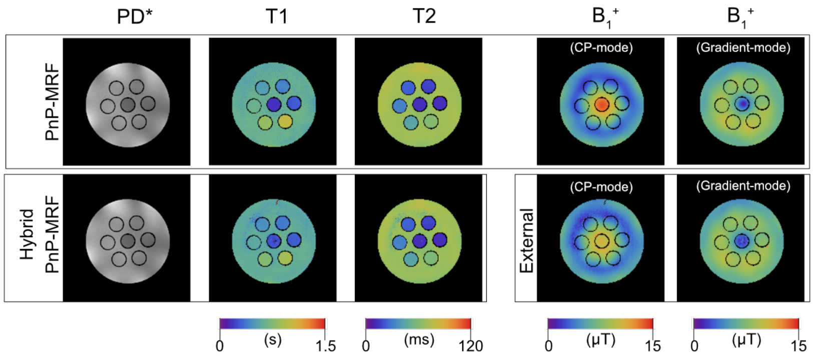

Supplementary Figure 2: Comparison of PnP-MRF and Hybrid-PnP-MRF. Top row: results from a PnP-MRF measurement of a phantom at 7 Tesla obtained using the comprehensive simultaneous reconstruction of PD, T1, T2 and B_1^+. Bottom row: results from a Hybrid-PnP-MRF measurement where the B_1^+ maps were first measured using a traditional fast B_1^+ mapping technique, which were than used to constrain the dictionary matching process. *Without receive sensitivity correction.

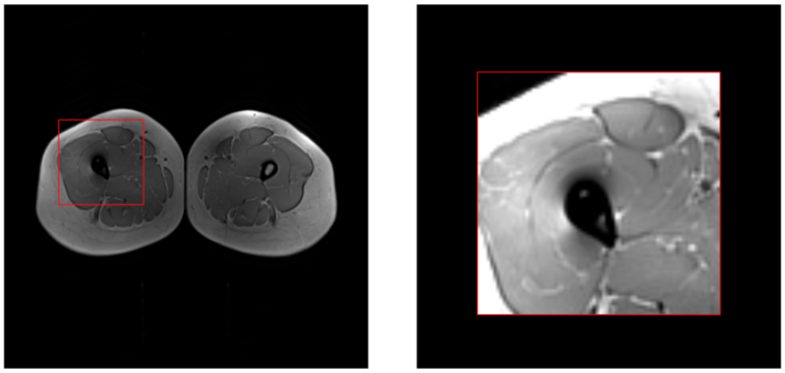

Supplementary Figure 3: A turbo spin echo image, traditionally classified as PD weighted. The sequence parameters were: TR = 4 s, TE = 8.4 ms, turbo factor = 8, no inversion, 320x320 matrix size,1.5x1.5mm^2 in-plane resolution and 5 mm slice thickness. Left: the full field of view. Right: enlargement of the area marked by the red frame in the figure to the left.

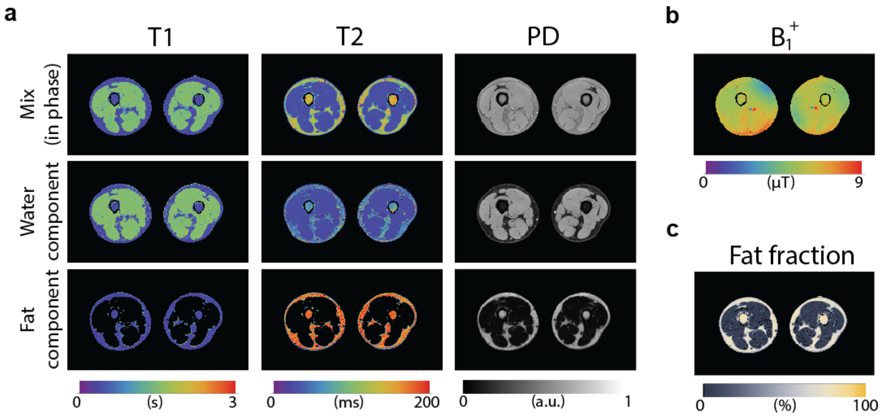

Supplementary Figure 4: Separation of fat & water components in each voxel based on the chemical shift effect. (**a**) Quantitative maps measured with the fat and water signal in phase (top row), the water component obtained by adding the in-phase and out-of-phase fingerprints prior to dictionary matching (middle row), and the fat component obtained by subtracting the fingerprints prior to matching (bottom row) (**b**) Heterogeneous RF field distribution produced by the anatomy specific EP-mode. (**c**) Map of the fat fraction obtained by taking the ratio between fat PD and total PD signal (fat + water). For a quantitative comparison to published literature values, see Supplementary Table 1.

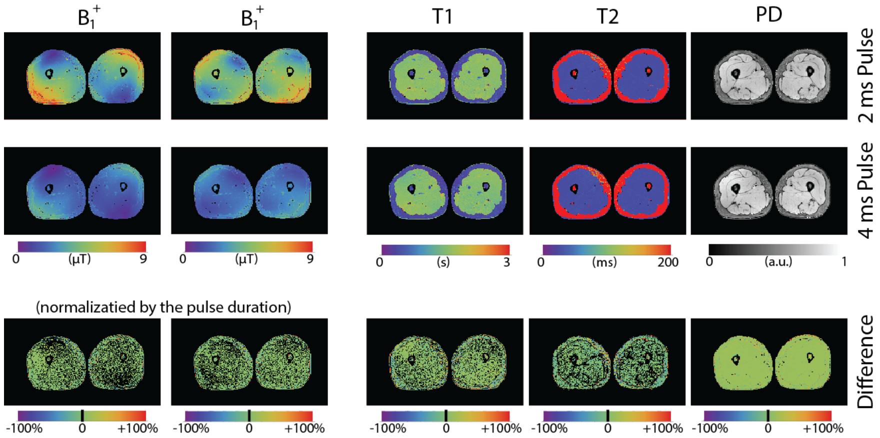

Supplementary Figure 5: Multi-parametric maps obtained using 2 different RF pulse durations in the PnP-MRF sequence. Both PD-maps show the same contrast between adipose and muscle tissue. In this case there is contrast between the adipose and muscle tissue due to prolonged TE (3.7ms, fat and water components 180 out-of-phase) compared to Fig. 6 (2.3ms, fat and water components in-phase).

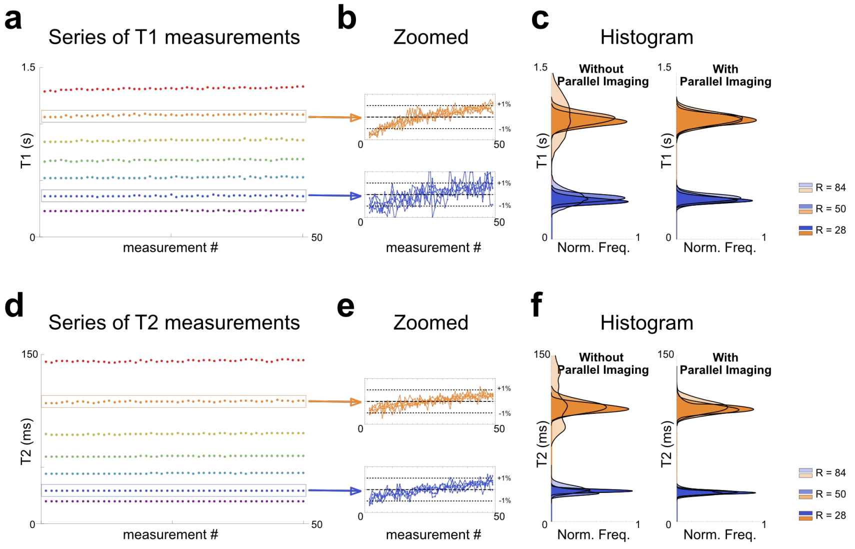

Supplementary Figure 6: Precision estimates derived from 50 repetitions of a PnP-MRF measurement. Three different acceleration factors (R = 84, R = 50 and R = 28) were evaluated (total of 150 measurements). (**a** & **d**) The T1 and T2 values measured in each of the 7 compartments in the phantom (averaged over a $\approx$ 100 voxel ROI). (**b** & **e**) The T1 and T2 values measured in two representative compartments (orange and blue line). Regardless of the acceleration factor or whether or not parallel imaging was used, the variation between measurements was less than $\pm2\%$ (in all 7 samples). (**c** & **f**) Histogram showing the frequency of each T1 or T2 value measured in the same two exemplary phantom compartments using all different acceleration rates and reconstruction methods (including all voxels without averaging over the ROI). Without parallel imaging, a clear decrease in variation can be seen with decreased acceleration factors (R = 84 vs R = 28, see also Supplementary Table 3). With parallel imaging, the variation among all samples is more subtle (see also Supplementary Table 2). Nevertheless, the same trend can be observed.

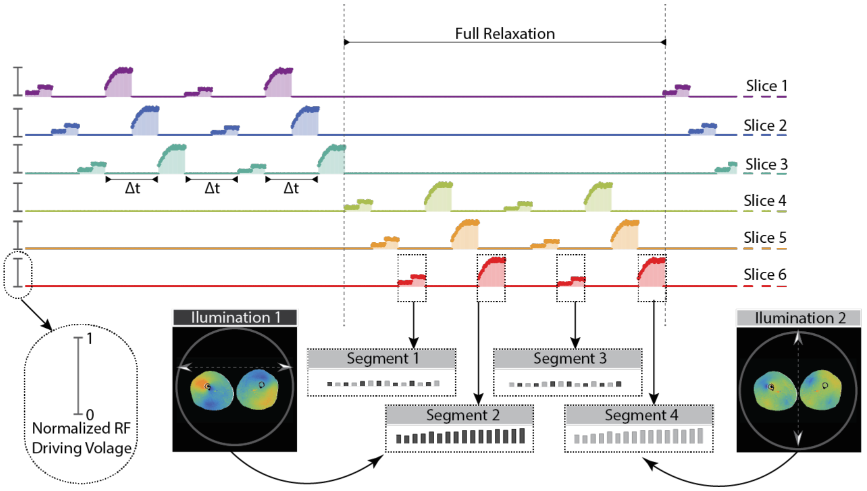

Supplementary Figure 7: Schematic overview of the PnP-MRF sequence. In this case only 6 slices are shown, the minimal number needed to fill all the delay times. The delay time Δt is defined as 240xTR, such that during this time two additional RF train segments can be played on different slices.

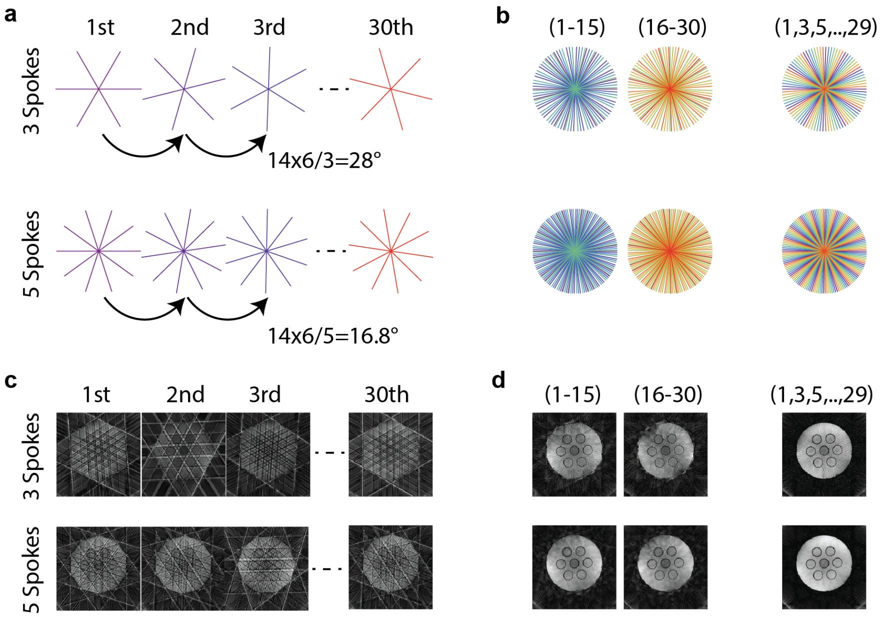

Supplementary Figure 8: Schematic overview of the radial sampling pattern for two different acceleration factors. Data are taken from the phantom validation data set. 3 and 5 spokes correspond to an acceleration factor of 84 and 50, respectively. (**a**) Sampling distribution for subsequent exposures using 3 and 5 spokes. (**b**) Superposition of the radial samples acquired during the first 15 exposures, the second 15 exposures, and the first 15 odd-numbered exposures, respectively. (**c**) Raw images (using direct nuFFT) corresponding to the sampling distributions shown in **a**. (**d**) The images obtained using direct nuFFT after combining the first 15, second 15, and first 15 odd-numbered exposures, respectively.

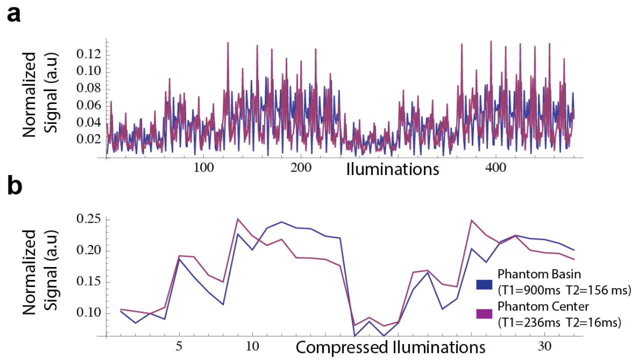

Supplementary Figure 9: Fingerprints measured in two different locations of the phantom (acquired with 3 spokes, acceleration factor 84). (**a**) The absolute of the normalized fingerprint before compression. (**b**) The absolute of the normalized compressed fingerprints.

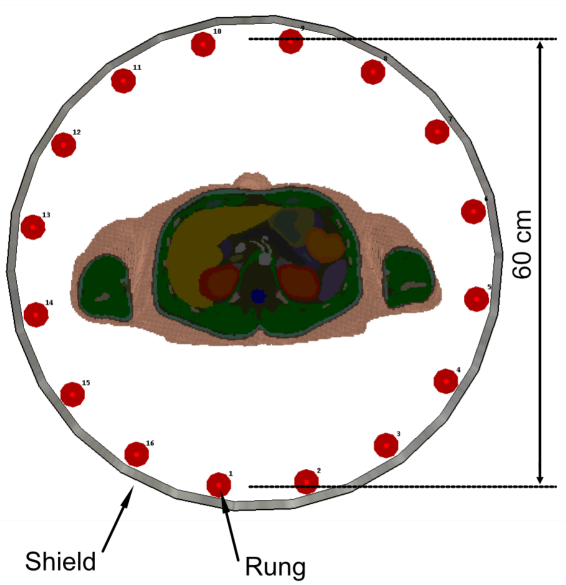

Supplementary Figure 10: Coil and human body model used in the electro dynamic simulations.

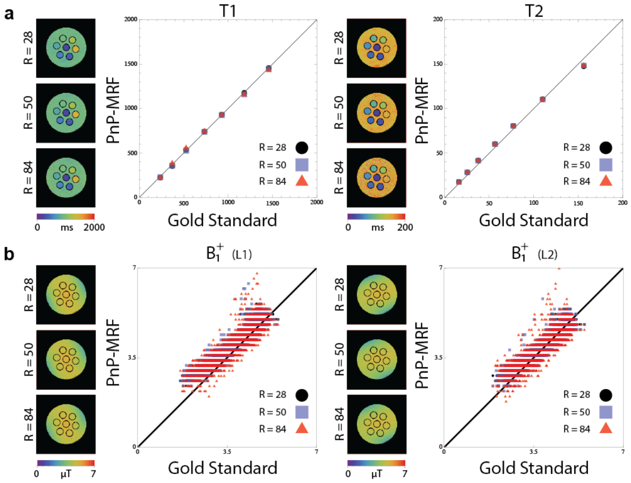

Supplementary Figure 11: Validation of PnP-MRF without parallel imaging. (**a**) The mean T1 & T2 value across each sample in milliseconds at different acceleration factors (R). (**b**) Scatter plots of the RF field amplitudes in μT at different acceleration factors.

Supplementary Table 1: Fat and water volume fractions measured in the leg.

**Study**	**Component**	**Muscle**			**Adipose**		
		T1(ms)	T2(ms)	%	T1(ms)	T2(ms)	%
**Gold, et al**	Mix	1420±38	32±2		371±8	133±4	
**This study**	Mix	1499±56	26±1		323±57	126±10	
**This study**	Fat	NA	NA	4	317±54	171±11	76
	Water	1512±52	26±1	96	396±71	54±10	24
**Thomas**	Fat						84
	Water						13
	Protein						3
**Tavichakorntrakooll, et al**	Fat			0			
	Water			80			
	Protein			20			

The T1, T2 and fat and water volume fractions measured in the legs of a asymptomatic volunteer compared to literature values. In this table, Mix indicates T1 and T2 values measured with the fat and water signal in-phase. When separated, the volume fraction of the fat and water components is indicated as a percentage of the total volume.

Gold, G.E., Han, E., Stainsby, J. & Wright, G. Musculoskeletal MRI at 3.0 T: relaxation times and image contrast. *A.J.R. Am. J. Roentgenol.* **183**, 343–351 (2004).

Thomas, L.W. The Chemical Composition of Adipose Tissue of Man And Mice. *Exp. Physiol.* **47**, 179–188 (1962).

Tavichakorntrakool, R., et al. K+, Na+, Mg2+, Ca2+, and water contents in human skeletal muscle: correlations among these monovalent and divalent cations and their alterations in K+-depleted subjects. *Transl. Res.* **150**, 357–366 (2007).

Supplementary Table 2: The T1 and T2 values measured in a 7-compartment phantom

	**Method**	**Time***	**#1**	**#2**	**#3**	**#4**	**#5**	**#6**	**#7**
T2 (ms)	Gold Standard	3h	16±1	25±1	38±2	56±2	77±2	110±1	156±1
	PnP (R = 28)	21s	16±1	26±2	39±2	58±2	78±3	110±4	153±6
	PnP (R = 50)	12s	16±1	26±2	38±2	57±3	78±3	110±6	153±7
	PnP (R = 84)	7s	16±1	26±2	39±2	57±3	78±3	110±5	154±7
T1 (ms)	Gold Standard	2h 30m	236±32	370±31	526±41	733±41	929±57	1181±51	1458±69
	PnP (R = 28)	21s	230±16	370±28	524±38	733±35	933±35	1209±47	1520±53
	PnP (R = 50)	12s	235±15	371±31	512±43	730±39	928±37	1193±52	1507±60
	PnP (R = 84)	7s	238±19	396±43	552±31	738±32	944±37	1197±50	1514±51

* PnP-MRF acquires both T1 and T2 simultaneously, whereas the gold standard measures only one at a time. The total scan time needed to obtain the gold standard for both T1 and T2 was 5h 30m.

The T1 and T2 values measured in a 7-compartment phantom compared to the gold standard. In this table, our PnP-MRF approach (one single measurement) is indicated by the abbreviation PnP. The scan time indicates the time needed per slice measured.

Supplementary Table 3: The T1 and T2 values measured without parallel imaging

	Method	Time*	#1	#2	#3	#4	#5	#6	#7
T2 (ms)	Gold Standard	3h	16±1	25±1	38±2	56±2	77±2	110±1	156±1
	PnP (R = 28)	21s	18±2	28±2	42±2	60±2	81±3	111±5	147±7
	PnP (R = 50)	12s	18±2	29±2	41±2	60±3	81±5	110±8	148±11
	PnP (R = 84)	7s	18±2	30±5	43±5	61±5	81±8	111±13	149±24
T1 (ms)	Gold Standard	2h 30m	236±32	370±31	526±41	733±41	929±57	1181±51	1458±69
	PnP (R = 28)	21s	223±15	352±34	535±47	741±40	930±46	1176±51	1455±52
	PnP (R = 50)	12s	226±18	357±51	525±70	737±58	927±52	1159±72	1445±77
	PnP (R = 84)	7s	227±26	392±82	565±81	753±71	938±76	1165±92	1439±121

* PnP-MRF acquires both T1 and T2 simultaneously, whereas the gold standard measures only one at a time. The total scan time needed to obtain the gold standard for both T1 and T2 was 5h 30m.

The T1 and T2 values measured in a 7-compartment phantom compared to the gold standard. In this table, the PnP-MRF fingerprints (one single measurement) were reconstructed without parallel imaging. The abbreviations and scan parameters are the same as in Supplementary Table 2.

Supplementary Note 1: Integrated vs external B_1^+ estimation

The proposed PnP-MRF implementation simultaneously estimates both the B_1^+ field distributions and the tissue specific properties, PD, T1 and T2 in one comprehensive reconstruction. Compared to the traditional MRF approach, the PnP-MRF dictionary contains 2 additional dimensions (one for each coil-mode). Since all these properties (PD, T1, T2, B_1^+) are entangled into the fingerprint, all these dimensions must be searched during the matching process. Not only to identify the B_1^+ field amplitude, but also find the correct PD, T1 and T2.

Alternatively, one could imagine a hybrid approach were the B_1^+ fields are first measured using a traditional B_1^+ mapping sequence. Such externally derived B_1^+ maps could than be used to constrain the cross-section of the dictionary that needs to be searched and could theoretically help improve the matching process. However, in practice, traditional B_1^+ mapping techniques are only accurate within a relatively narrow dynamic range. Moreover, many of the faster B_1^+ mapping strategies are optimized for specific range of T1 values. Thus, if the T1 values are too short [1] or the B_1^+ exceed the optimal range [1, 2, 3] external maps may introduce small systematic errors into the multi-parametric maps (PD, T1, and T2) obtained with such a hybrid approach.

To investigate these effects, we used our 7 Tesla MRI system (Magnetom 7T, Siemens, Germany) to perform such a Hybrid-PnP-MRF experiment. The same 8-channel dipole array head-coil and phantom was used as described earlier. The pre-saturation turbo-FLASH sequence was used to obtain a set of external B_1^+ measurements [1]. To avoid unwanted slice profile effects a rectangular pre-saturation pulse was used. The sequence parameters were as follows: 1 slice, 4 averages, TR = 10s, inter echo time (ITE) = 3ms, 160x160 matrix, in-plane resolution 1.5x1.5mm^2, 5.0 mm slice thickness, total scan time 4min.

Comparing the results obtained with PnP-MRF and the Hybrid-PnP-MRF approach, small, but noticeable, artifacts appear in the T1 and T2 maps in the areas corresponding to relatively low and high B_1^+ (Supplementary Figure 2 Top vs Bottom row).

Supplementary Note 2: Magnetization transfer effects

Although the absence of fat-muscle contrast in the quantitative in vivo PD-map in Fig. 6 is not entirely unexpected, it is notably different from what is typically observed in TSE-based clinical PD-weighted images (Supplementary Figure 3). Magnetization transfer (MT) is often linked to contrast changes in fast imaging sequences [4, 5, 6, 7]. Changing the RF pulse duration in the DESPOT sequence, for instance, can alter the observed T1 and T2 values [6, 8].

To rule out an MT bias in our PD-maps, we performed additional experiments with two different RF pulse durations (asymptomatic volunteer 27Y, F). The parameters for our PnP-MRF sequence were as follows: 18 slices, TR/TE = 7.5/3.7ms, RF time bandwidth product 3, RF pulse duration 2 or 4 ms, 240x240 matrix, 2.0x2.0mm^2 in-plane resolution, 5mm slice, acceleration factor R $\approx$ 42 (9 spokes per time point, $\pm$35s per slice).

Due to the factor of 2 difference in RF pulse duration, the B_1^+ field amplitude measured using the 4ms pulse duration is half of that using the 2ms pulse (Supplementary Figure 5). When the field maps are normalized by the pulse duration, no significant changes can be discerned. Likewise, looking at the tissue properties, no significant changes in T1, T2, and PD were observed.

Supplementary Note 3: Proton density in adipose and muscle tissue

According to [9], adipose tissue contains about 20% water and 80% fat. Based on the results from [10], and scaling the different acid contributions by their molecular weights we find 0.126 mol H/g pure fat. Water, on the other hand, contains 0.111 mol H/g. By weight, the fat/water fraction in adipose tissue is 85%/15%, which results in a proton density of 0.124 mol H/g. According to [11], muscle contain 80% water, and the residual composition of muscle is mostly proteins, the most abundant of which is actin (molecular weight of 0.0692 mol H/g). Under the assumption that the mean proton density in all these proteins can be approximated using actin, we find 0.80 x 0.111 + 0.20 x 0.0692 = 0.103 mol H/g. The PD-map measured using our PnP-MRF approach quantifies the relative PD between tissues per unit volume. Muscle has a density of 1.06 g/mL, which results in a PD of 1.06 x 0.103 = 0.109 mol H/mL. Adipose tissue has a density of 0.92g/mL, which results in a similar PD of 0.92 x 0.124 = 0.114 mol H/mL.

Supplementary Note 4: Separation of fat & water signals in MRF

The results shown in Fig. 6 were acquired using the shortest possible echo time (TE) and repetition time, resulting in a total scan time of approximately 28s per slice. Prolonging the TE from 2.4 ms to 3.7 ms increases the scan time by approximately 20%, but allows the fat and water components to accumulate a 180 phase difference [12]. When both echo times are acquired, addition or subtraction of fingerprints measured at those times results in separate fat and water fingerprints.

Using the same setup as described for the in-vivo experiments, additional images were acquired using two different echo times to separate the fat and water signals measured in an asymptomatic volunteer (26, M). The parameters for our PnP-MRF sequence were as follows: 18 slices, TR/TE1/TE2 = 8.0/2.4/3.7ms, RF time bandwidth ratio 3, RF pulse durations 2 ms, 240x240 matrix, 2.0x2.0 mm^2 in-plane resolution, 5 mm slice, acceleration factor R $\approx$ 36 (10 spokes per uncompressed time point, $\pm$36s per slice). The phase difference between the two echoes was used to determine the relative sign between the two fingerprints. To avoid a bias due to susceptibility-related phase variations, a threshold was set at 90. In other words, if the absolute value of the phase difference between the two TEs exceeded 90 degrees, fat was assumed to be the dominant component.

After separation, the T1, T2, and PD of water (Supplementary Figure 4a, middle row) and fat (Supplementary Figure 4a, bottom row) components can be quantified individually in each voxel. In this asymptomatic volunteer, the presumably healthy muscle tissue contains little or no fat (Supplementary Figure 4c). However, pathological conditions, such as Limb-Girdle Muscular Dystrophy, may be characterized by fat infiltration into the muscle.

The quantitative results are summarized in Supplementary Table 1. These preliminary results match well with the mass spectrometry data presented in Supplementary Note 2. However, they slightly overestimate the fat fraction compared to that derived from traditional MR methods such as the IDEAL method [13]. One possible explanation for this discrepancy could be that our dual echo approach does not take into account the full spectral complexity of the fat signal. Further work is needed to explore the origins of this discrepancy, and improve the robustness to B0 variations.

Supplementary Note 5: Direct nuFFT vs parallel imaging

Traditionally, extreme acceleration factors, like those used in this work, result in insurmountable aliasing artifacts (which appear as streaks in radially-acquired data). In the MR fingerprinting framework, however, these incoherent artifacts add a noise-like modulation to the fingerprint, which has a relatively benign effect on the reconstruction process. This can easily be understood in terms of temporal frequencies. The frequency with which the sharp undersampling artifacts appear is related to the inter-echo time, whereas the tissue-dependent relaxation components result in relatively smooth decay curves. Consequently, even without parallel imaging techniques [14, 15, 16], the reconstruction process, in which each measured fingerprint is compared to the artifact-free entries in a pre-simulated dictionary, naturally filters out the high frequency perturbations introduced by the undersampling artifacts (Supplementary Figure 11, & Supplementary Table 3). Nevertheless, when the acceleration factor is too high, streak-like artifacts can be seen shining through (Supplementary Figure 11, $R = 84$). The different sensitivity profiles produced by an array of receive coils can be leveraged to estimate missing points in k-space [14, 15, 16]. Using an iterative reconstruction framework [17], the streak-like artifacts produced by an undersampled radial sampling pattern can be removed.

However, a requirement of the classical parallel imaging paradigm is that the number of receive elements N is equal to or larger than the acceleration factor. In our sequence, the R used to measure each individual time point in the fingerprint easily exceeds N. Therefore image reconstruction corresponds to finding a solution of an underdetermined system of equations and additional constraints have to be introduced to impose a certain structure on the solution. Commonly used choices are Tikhonov regularization [18] or nonlinear l1 sparsity promoting terms supported by the framework of compressed sensing [19]. Another popular approach is to stop the iterative method that is used to solve the system of equations before convergence is achieved. Essentially these constraints allow one to trade off non-uniform noise amplification as described by the g-factor of the coil array [15] against residual aliasing, image blurring and introduction of compression-type artifacts depending on the type of regularization and the k-space acquisition trajectory employed.

Our implementation of iterative SENSE [17] uses the method of conjugate gradients with 5 iterations. Applying the compression in k-space prior to reconstruction alleviates blurring effects by reducing the effective acceleration factor to $R* = R/15$ (where 15 is the compression factor used in this work). Nevertheless, as $R*$ approaches N, signs of image blurring will start to appear in the parametric maps (In our case this typically happens around $R > 50$).

Supplementary References

[1] Chung, S., Kim, D., Breton, E. & Axel, L. Rapid B_1^+ mapping using a preconditioning RF pulse with TurboFLASH readout. *Magn. Reson. Med.* **64**, 439–446 (2010).

[2] Yarnykh, V.L. Actual flip-angle imaging in the pulsed steady state: A method for rapid three-dimensional mapping of the transmitted radiofrequency field. *Magn. Reson. Med.* **57**, 192–200 (2007).

[3] Nehrke, K. & örnert, P. DREAM-a novel approach for robust, ultrafast, multislice B1mapping. *Magn. Reson. Med.* **68**, 1517–1526 (2012).

[4] Bernstein, M.A., King, K.F. & Zhou, X.J. *Handbook of MRI pulse sequences.* (Academic Press 2004).

[5] Boulant, N. T1 and T2 effects during radio-frequency pulses in spoiled gradient echo sequences. *J. Magn. Reson.* **197**, 213–218 (2009).

[6] Bieri, O., & Scheffler, K. On the origin of apparent low tissue signals in balanced SSFP. *Magn. Reson. Med.* **56**, 1067–1074 (2006).

[7] Gloor, M., Schefflerm K. & Bieri, O. Quantitative magnetization transfer imaging using balanced SSFP. *Magn. Reson. Med.* **60**, 691–700 (2008).

[8] Gloor, G., Scheffler, K. & Bieri, O. Nonbalanced SSFP-based quantitative magnetization transfer imaging. *Magn. Reson. Med.* **64**, 149–156 (2010).

[9] Thomas, L.W. The Chemical Composition of Adipose Tissue of Man And Mice. *Exp. Physiol.* **47**, 179–188 (1962).

[10] Malcom, G.T., et al. Fatty acid composition of adipose tissue in humans: differences between subcutaneous sites. *Am. J. Clin. Nutr.* **50**, 288–291 (1989).

[11] Tavichakorntrakool, R., et al. K+, Na+, Mg2+, Ca2+, and water contents in human skeletal muscle: correlations among these monovalent and divalent cations and their alterations in K+-depleted subjects. *Transl. Res.* **150**, 357–366 (2007).

[12] Dixon, W.T. Simple proton spectroscopic imaging. *Radiology* **153**, 189–194 (1984).

[13] Reeder, S.B., et al., Water/Fat Separation With IDEAL Gradient-EchoImaging *J. Magn Reson Imagn* **25**, 644–652 (2007).

[14] Sodickson, D.K. & Manning, W.J. Simultaneous acquisition of spatial harmonics (SMASH): Fast imaging with radiofrequency coil arrays. *Magn. Reson. Med.* **38**, 591–603 (2007).

[15] Pruessmann, K.P. & Weiger, M. SENSE: sensitivity encoding for fast MRI. *Magn. Reson. Med.* **42**, 952–962 (1999).

[16] Griswold, M.A., et al. Generalized autocalibrating partially parallel acquisitions (GRAPPA). *Magn. Reso. Med.* **47** 1202–1210 (2002).

[17] Pruessmann, K.P., Weiger, M,. Börnert, P., & Boesiger, P. Advances in sensitivity encoding with arbitrary k-space trajectories. *Magn. Reson. Med.* **46**, 638–651 (2001).

[18] Lin, F., Kwong, F.K., Belliveau, J.W. & Wald, L.L. Parallel imaging reconstruction using automatic regularization. *Magn. Reson. Med.* **51**, 559–567 (2004).

[19] Lustig, M., Donoho, D. & Pauly, J.M. Sparse MRI: The application of compressed sensing for rapid MR imaging. *Magn. Reson. Med.* **58**, 1182–1195 (2007).
